# Factors associated with vulvar cancer from 2005 to 2021 in Togo, sub-Saharan Africa

**DOI:** 10.1186/s12905-023-02669-6

**Published:** 2023-09-26

**Authors:** Tchin Darré, Bagassam Sama, Toukilnan Djiwa, Whitney E.D. Afantodji-Agbeti, Mayi Bombone, Yendoubé Kambote, Panakinao Simgban, Bingo K M’Bortche, Baguilane Douaguibe, Koffi Amégbor, Mazamaesso Tchaou, Abdoul-Samadou Aboubakari, Bayaki Saka, Gado Napo-Koura

**Affiliations:** 1 Department of Pathology, University Teaching Hospital of Lomé, Lomé, Togo; 2 Department Obstetrics and Gynecology, University Teaching Hospital of Lomé, Lomé, Togo; 3 Department of Imaging, University Teaching Hospital of Lomé and Kara, Lomé, Togo; 4 Department of Dermatology, University Teaching Hospital of Lomé, Lomé, Togo

**Keywords:** Cancer, Vulva, Human papilloma virus, Squamous cell carcinoma, Togo

## Abstract

**Background:**

vulvar cancer, once predominantly diagnosed in older women, is increasingly being diagnosed in younger individuals, due to Human Papillomavirus (HPV) infection. Our study aimed to describe the epidemiological and histopathological aspects of vulvar cancer in Togo and its associated factors.

**Methods:**

This was a cross-sectional study, conducted on vulvar cancer cases histologically diagnosed at the Pathological Laboratory of Lomé over a period of 17-years (2005–2021). Parameters investigated included age, occupation, risk factors, sample nature, macroscopic tumor aspects, histological types, therapeutic intervenions, and prognostic outcomes.

**Results:**

A total of 32 cases of vulvar cancer were collected, yieding an annual frequency of 1.88 cases. The average age of the patients was 48±14.12 years with extremes of 27 years and 82 years. Housewives accounted for the largest proportion of cases (37.5%). Among the 32 cases, 27 had identifiable risk factors, with HPV infection being the most prevalentr (33.3%). The ulcero-budding aspect was most frequently observed, and squamous cell carcinoma was the most common histological type, with the majority being well differentiated (89.3%). Statistically significant associations were found between risk factors and histological types, risk factors and degrees of differentiation, as well as between histological types and good differentiation of vulvar cancers. The 3-year survival was estimated at 78.13%.

**Conclusion:**

The incidence of vulvar cancer is increasing in Togo, particularly among young, primarily due to HPV infection.

## Background

Vulvar cancer is a malignant tumor proliferation that originates from various structures of the vulva [1]. Cancers, including vulvar cancer, are part of the broader category of non-communicable diseases and pose a significant public health concerndue to their increasing incidence and high mortality rates. Vulvar cancer ranks as the fouth most common gynecological cancer, following cervical, uterine, and ovarian cancers [2, 3]. It accounts for approximately 2–5% of all gynecological cancers and 1% of all cancers diagnosed in women [2, 4]. In 2020, there were 45,240 newly diagnosed cases of vulvar cancer, representing 0.2% of all cancers in terms of incidence, and it resulted in 17,427 deaths [5]. In the USA, vulvar cancer constitutes 5% of all female cancers [6]. In France, its incidence ranges from 1 to 2/100,000 women [7]. In sub-Saharan Africa, vulvar cancer is often diagnosed at advanced stage. It constitutes 2.7% of cancers in women in Senegal, 4% in Cameroon and 2.21% in Gabon [5, 7, 8]. Traditionally, vulvar cancer has been associated with postmenopausal women, typically occuring in the 65–70 age group [9–11], partly due tovulvar lichen sclerosis lesions commonly found in older postmenopausal women [12, 13]. However, there has been a shift over time, with an increasing incidence of vulvar cancer in young women [7, 9, 11]. Several risk factors have been implicated in this trend, including persistent HPV infection, smoking, early age at first sexual intercourse, multiple sexual partners, sexually transmitted infections such as Herpes Simplex and Papilloma Virus, immunosuppression (often associated with HIV), vulvar inflammatory diseases, family history of vulvar cancer, personal history of cervical cancer, and low socioeconomic status [9, 14, 15]. In a 2017 study on cancer epidemiology in Togo, Darré et al. reported a 0.6% frequency of vulvar cancers [16]. However, this study did not specifically focus on vulvar cancer and did not consider risk factors associated with this cancer. Therefore, our research aims to provide insights into epidemiological and histopathological characteristics of vulvar cancer in Togo, with a specific focus on associated risk factors.

## Methods

### Study design

This study was a cross-sectional descriptive analysis of vulvar cancer cases histologically diagnosed in the pathological anatomy laboratory of the Lomé University Hospital over a period of 17 years (2005–2021). Togo, with a land area of 56,600 km2 and an estimated population of 7,200,000, is situated between Ghana to the west, Benin to the east, and Burkina Faso to the north [16].

### Study population

The study included all cases of histologically confirmed vulvar cancer. These cases were collected from the laboratory’s records. The study material comprised biopsies and surgical specimes fixed in 10% formalin and processed using standard histology techniques. The variables studied included socio-demographic data (age, occupation, year of diagnosis), risk factors, symptom types, pathological data (sample type, histological type of the lesion), therapeutic interventions, and prognostic outcomes.

All patients diagnosed with vulva squamous cell carcinoma underwent HPVscreening using PCR. The PCR analysis was conducted using DNA extracted from paraffin-embedded tissue samples obtained from these patients. The classification of cancer cases was based on the 2017 UICC classification (UICC, 8è edition). Subsequently, statistical analysis and data processing were performed to analyze the collected information [15].

### Data processing and analysis

Univariate and multivariate logistic regression analyses were conducted to identify factors associated with delayed consultation. The independent variable was the long consultation delay coded 1 if yes and 0 if not. When the independent variable was statistically associated with the dependent variable during the univariate analysis with a degree of significance threshold was set at 0.05, it was introduced into the initial model. The top-down step-by-step procedure was used for final model selection. It consisted of including the variables chosen in the initial model. Univariate analysis was used to estimate the odds ratio (OR) and its 95% confidence interval. The multivariate analysis made it possible to estimate the adjusted odds ratio (aO) and its 95% confidence interval for each variable retained.

## Results

### Epidemiological data

Table 1 summarizes the socio-demographic characteristics of the patients. We collected 32 cases of vulvar cancer, resulting in an annual frequency of 1.88 cases. The average age of our patients was 48 ± 14.12 years with extremes of 27 and 82 years. Eleven women (34.3%) were aged between 35 and 45 years. Twelve (12) of our patients were housewives representing 37.5%, followed by 7 farmers (21.9%). Twenty-seven patients (84.4%) had identifiable risk factors, with HPV infection being the most prevalent (33.3%).

**Table 1:** Summarizes the socio-demographic characteristics of the patients

	Number	%
**Age (years)**		
[25–35]	3	9.4
[35–45]	11	34.3
[45–55]	10	31.25
[55–65]	3	9.4
[65–75]	3	9.4
[75–85]	2	6.25
**Profession**		
Housewife	12	37.5
Farmer	7	21.9
Liberal profession	7	21.9
Civil servant	4	12.5
Student	2	6.2
**Risk factors**		
Yes	27	84.4
HPV* infection	9	33.3
Multiple sexual partners	6	22.2
Family history of vulvar cancer	5	18.5
Personal history of cervical cancer	5	18.5
Dysplastic lesions	5	18.5
Smoking	3	11.1
History of cervical cancer	1	3.7

HPV*= Human Papilloma Virus.

### Pathological data

**Fig. 1:**
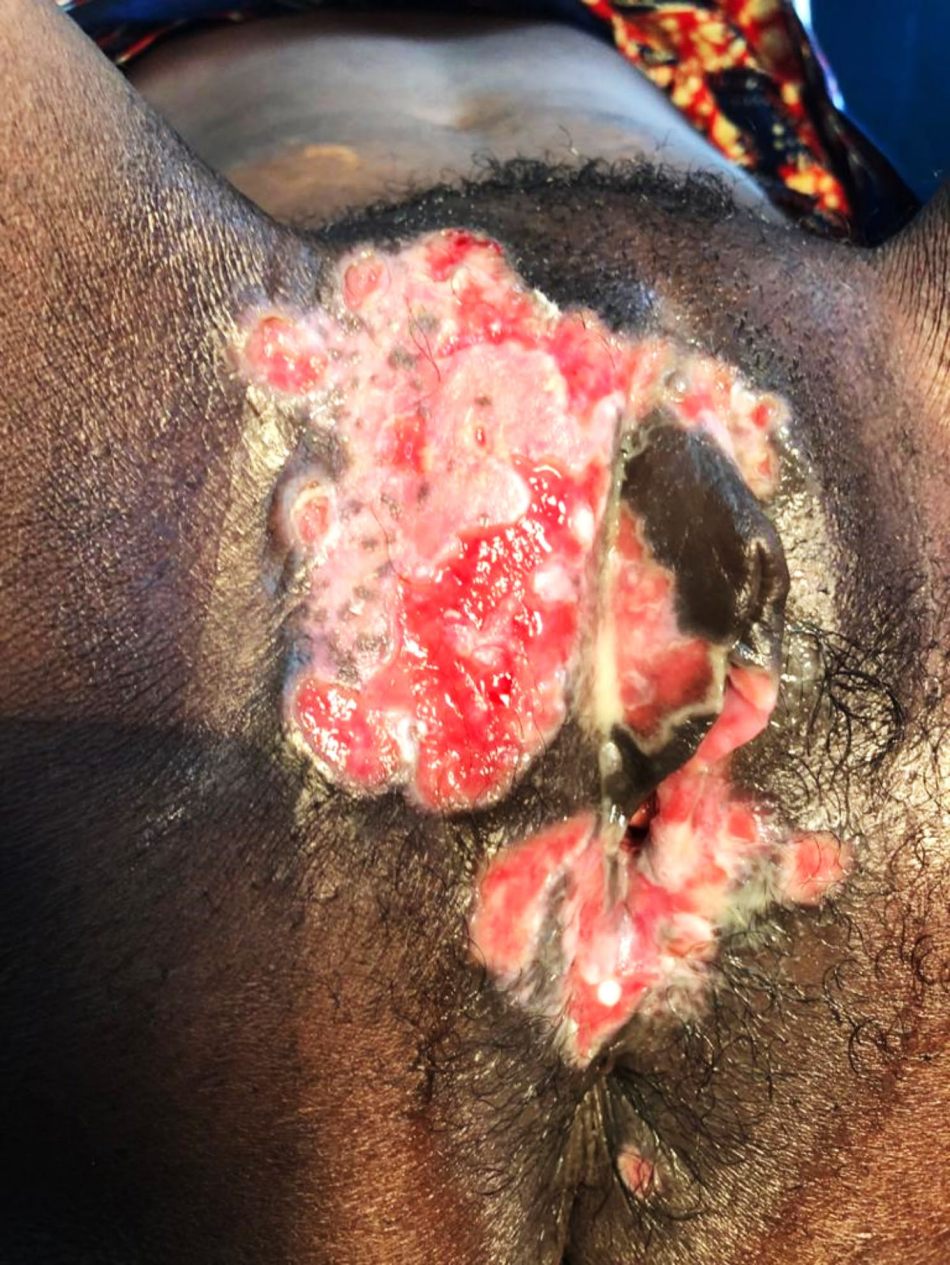
Vulvar ulcero-budding tumor

**Fig. 2:**
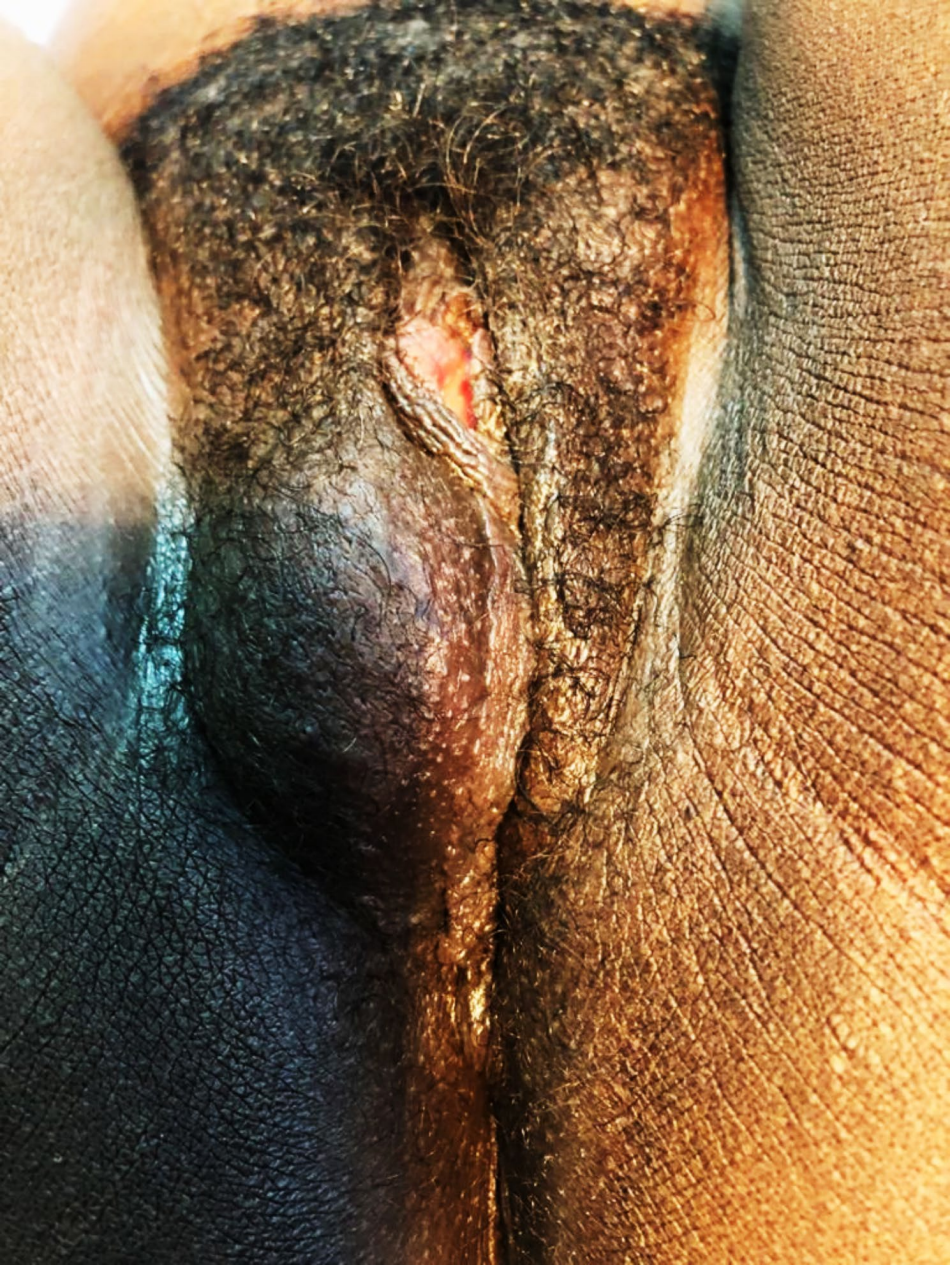
Nodular infiltrating vulvar tumor

The diagnosis exclusively based on biopsies. The most frequently observed macroscopic aspects were ulcero-budding forms (10 cases; 31.3%), followed by the ulcerated form with 8 cases (25%). Table 2 and Figs. (1, 2) summarize the macroscopic aspects of patients’ vulvar cancers. Histologically, squamous cell carcinoma represented 22 cases (68.75%), followed by adenocarcinoma with 6 cases (18.75%) and melanoma with 4 cases (12.5%). All adenocarcinomas in our study were well differentiated. Among squamous cell carcinomas, 19 of 22 cases (86.4%) were differentiated, while 3 cases (13.6%) were poorly differentiated. Table 3 highlights the association between risk factors and histological types of vulvar cancers, with a statistically significant association observed between HPV infection and the histological type of vulvar cancer (p-value = 0.0041). We found data on the differentiation of histological types in 28 patients. The cross between the risk factors and the differentiation of the histological types of vulvar cancers revealed a statistically significant association HPV and the differentiation (p-value = 0.0068), as depicted in Table 4. In multivariate analysis, there is a statistically significant association between histological type and good tumor differentiation (aOR = 1; p-value = 0.001) (Table 5).

**Table 2:** Macroscopic aspects of the vulvar cancer

	Value	%
**Ulcer-bourgeoning tumor**	**10**	**31.3**
Ulcerated tumor	8	25
Infiltrating tumor	5	15.6
Nodular tumor	5	15.6
Budding tumor	4	12.5
**Total**	**32**	**100**

**Table 3:** Univariate analysis of the different factors and histological types of vulvar cancer

	Histological types				
	squamous cell carcinoma	Adenocarcinoma	Melanoma	Total	p-value
	n (%)	n (%)	n (%)	n (%)	
HPV* infection	5(55.6)	4(44.4)	0(0)	**9(100)**	**0.041**
Multiple sexual partners	4(66.7)	0(0)	2(33.3)	**6(100)**	0.1491
Family history of vulvar cancer	3(60)	0(0)	2(40)	**5(100)**	0.139
Personal history of cervical cancer	4(80)	1(20)	0(0)	**5(100)**	1
Dysplastic lesions	3(60)	2(40)	0(0)	**5(100)**	0.322
Smoking	1(33.3)	0(0)	2(66.7)	**3(100)**	0.051
Family history of cervical cancer	1(100)	0(0)	0(0)	**1(100)**	1

HPV*= Human Papilloma Virus.

**Table 4:** Univariate analysis of the different factors and degree of differentiation vulvar cancers

	Histological types			
	Well differentiated (25)	Poorly differentiated (3)	Total (28)	p-value
	n (%)	n (%)	n (%)	
HPV* infection	8(88.9)	1(11.1)	9(100)	**0.0068**
Personal history of cervical cancer	4(80)	1(20)	5(100)	0.22
Dysplastic lesions	5(100)	0(0)	5(100)	1.07
Multiple sexual partners	4(100)	0(0)	4(100)	0.94
Family history of vulvar cancer	3(100)	0(0)	3(100)	0.601
Smoking	1(100)	0(0)	1(100)	1.788
Family history of cervical cancer	1(100)	0(0)	1(100)	2.011

HPV*= Human Papilloma Virus.

**Table 5:** Multivariate analysis of the different factors involved, histological types and good cancer differentiation

	Univariate analysis					Multivariate analysis		
	Good differentiation							
	n/N	%	OR	95% CI	p-value	ORa	95% CI	p-value
**Age (years)**					0.23			
[25–35]	3 / 3	100	1					
[35–45]	9 / 10	90	0.62	0.45–2.77				
[45–55]	9 / 9	100						
[55–65]	2 / 2	100						
[65–75]	1 / 3	33.3	0.71	0.34–1.88				
[75–85]	1 / 1	100						
**Histological types**					**0.0004**			**0.001**
Adenocarcinoma	6 / 6	100	1			1		
Squamous cell carcinoma	19 / 22	86.4	0.74	0.25–0.96		0.55	0.4–0.81	
**Risk factors**					0.073			
Yes	22 / 24	91.7	1.39	1.24–1.92				
No	3 / 4	75	1					

### Therapeutic and prognostic data

Radiotherapy was delivered to 5 (15.63%) patients at a dose ranging from 60 to 70 Gy. Eight (8 cases; 25%) received chemotherapy based on cisplatin and 5-fluorouracil (5-FU).

After 3 years of follow-up; 7 (21.87%) patients died, 5 (15.63%) patients were lost to follow-up, 3 (9.37%) and 2 (6.25%) patients presented respectively with uterine and inguinal lymph node extension. None of the patients presented distant metastases. The 3-year survival was estimated at 78.13%.

## Discussion

### Epidemiological data

Vulvar cancer is the 4th most common type of gynecological cancer after cervical, endometrial and ovarian cancers. It is a rare entity that comprises approximately 6% of all malignant tumors of the female genital tract. The likelihood of developing vulvar carcinoma tends to with age [1]. Notably, there has been an upward trend in vulvar cancer incidence in recent decades, particularly among younger women. Persistent genital HPV (Human Papillomavirus) infection has been identified as a primary factor contributing to the development of vulvar neoplasia in this demographic [2]. In HIV-positive women, the presence of HIV-induced immunodeficiency plays a significant role in promoting HPV infection and its persistence. Consequently, this immunodeficiency makes these women more susceptible to developing genital cancers, including vulvar cancer [9]. Indeed, HIV-infected women control HPV infection much less well than uninfected women [10] and, among HIV-infected women, those with a CD4 + T-lymphocyte count of less than 200 cells. /mm^3^ are less likely to clear an HPV infection than women with a CD4 + T-lymphocyte count of between 200 and 500 cells/mm^3^ [9].

Our study revealed an annual frequency of 1.8 cases of vulvar cancer, reflecting its relatively low frequency. In Burkina Faso, Nayi Zongo et al. in 2016, reported an annual frequency of 8.4 cases of vulvar cancer [17]. The relatively low frequency in our series may be attributed to potential underestimation of cases, as some samples might not have reached the laboratory A study conducted by Amegbor K. et al. in Togo in 2011 found an annual frequency of 0.8 cases of vulvar cancer [18], which highlights the increasing prevalence of this cancer in our context.

The average age of the patients in our study was 48 ± 14.12 years with extremes of 27 years and 82 years. Historically, vulvar cancer is a relatively rare cancer, occurring most often in older women [19, 20]. However, over time, over time, there has been a noticeable shift with an increasing occurrence of vulvar cancer among younger women [7, 9, 11]. Several risk factors, such as persistent HPV infection, have been implicated in this changing trend [14, 15, 21].

### Pathological data

The majority of vulvar cancers are histologically classified as squamous cell carcinomas, with melanoma, adenocarcinoma of the Bartholin glands, or skin tumors being less common occurrences [14, 15]. These cancers often presents macroscopically budding, ulcerated lesions and rarely nodular [1, 19].

The ulcero-budding aspect was the most recorded aspect with 31.3%, followed by the ulcerated aspect with 25%. This aligns with findings from Nayi Zongo’s study, where the ulcero-budding aspect was also the most predominant, reported in 81% of cases [17]. Existing literature also supports the ulcero-budding aspect as the most common presentation of vulvar cancer [9, 11]. Squamous cell carcinoma was the predominant histological type with 68.8%. Squamous cell carcinoma consistently ranks as the most frequent histological type of vulvar cancer in the literature [5, 7, 9–11]. Squamous cell cancers, much like cervical and vaginal cancers, is preceded by lesions of the intraepithelial neoplasia type VIN ( vulvar intraepithelial neoplasia), The transformation of these lesions into invasive cancer occurs at a rate of approximately 5 to 10%, whether bowenoid papulosis, genital warts or Bowen’s disease [9, 11]. Among the risk factors associated with vulvar cancer in our context, only HPV infection demonstrated a statistically significant association with the histological type of vulvar cancers (p-value = 0.0041). Similar findings were reported by studies conducted by M. Van Beurden in the Netherlands and Henri Azaïs in France, both of which identified the involvement of HPV in the development of vulvar cancer [22, 23].

Recent research focused on vulvar skin epithelium has shown that there are changes in the expression of the P53 protein that may precede the appearance of vulvar intraepithelial carcinomas. This suggests that P53 mutations could be an initial event in the genesis of vulvar carcinoma [24]. Dynes McConell reported that mutations in the P53 protein are found in more than 50% of vulvar cancers, and that genital HPV infection increases the risk of the occurrence of P53 mutations [24].

### Therapeutic and prognostic data

Radiotherapy was delivered to 5 (15.63%) patients at a dose ranging from 60 to 70 Gy. Eight patients (25%) received chemotherapy based on cisplatin and 5-fluorouracil (5-FU).

The standard treatment for even small invasive carcinoma of the vulva was radical vulvectomy, which entails removing the primary tumor with a wide margin, followed by en-bloc resection of the inguinal lymph nodes and, in many cases,, pelvic lymph nodes [2]. This operation is said to have a high morbidity rate, with around 50% wound infections and post-operative complications [2]. For patients who are candidates for chemotherapy, chemoradiotherapy may be preferred, according to data in vulvar cancer [25]. Chemoradiotherapy is particularly recommended in cases involving anorectal, urethral, or bladder involvement, tumors attached to the bone, or lymph node involvement. The recommended chemotherapy agents include cisplatin, 5-FU, or mitomycin C, which are used in combination with radiation therapy. In some cases, surgery may be considered after chemotherapy and radiotherapy due to the reduction in tumor size achieved by this combination [26]. It’s worth noting that chemotherapy for vulvar cancer is often palliative and can be less effective due to the limited number of cases requiring this treatment. Therefore, there is currently no established standard treatment for such cases. Given the low response rate to conventional chemotherapies, there is increasing interest in exploring new biological agents like gefitinib and erlotinib. These agents, classified as reversible tyrosine kinase inhibitors, administered orally [27]. These enzymes are associated with the human epidermal growth factor receptor (EGFR), by inhibiting tyrosine kinase, gefitinib and erlotinib prevent EGFRs from stimulating uncontrolled cell growth that contributes to tumor growth. Gefitinib combined with trastuzumab has been studied in a human vulvar carcinoma cell line (A431) and appears to increase radiosensitivity [27].

Although the available data are fewer, patients who cannot undergo surgical treatment should receive primary radiotherapy [28]. The total radiation dose should be between 60 and 70 Gy, and the inguinal and pelvic regions should be treated bilaterally in case of positive lymph node involvement. Also, primary radiation therapy can be used preoperatively for women with advanced vulvar cancer. High rates of tumor shrinkage and complete responses at the time of surgery have been reported [28]. After 3 years of follow-up; survival was estimated at 78.13% in our patients.

The prognosis for patients with vulvar cancer is quite good when the treatment is given in time. Inguinal and/or femoral lymph node involvement is the most important prognostic factor for the survival of patients with vulvar cancer [29, 30]. Extracapsular growth of lymph node metastases, involvement of at least two lymph nodes and replacement of more than 50% of lymph nodes by tumor are predictive factors of poor survival [29, 31].

A immunohistochemical, p16 expression and HPV positivity are linked to a better prognosis, while p53 overexpression is linked to a worse prognosis; thus, biomarkers could help tailoring conventional treatment and follow-up [32]. The implications of PD-L1 positivity in reference to HPV status and prognosis are still not clear, even though pembrolizumab is part of available systemic therapies [32]. The role of tumor angiogenesis emerges through data on microvessel density, immunohistochemical VEGF staining and evaluation of serum VEGF concentrations. Few data exist on hormonal receptor expression, even though hormonal therapy showed great manageability [33].

The overall 5-year survival rate varies from 70 to 93% for patients whose nodes are negative and from 25 to 41% for those whose nodes are positive [31]. Other prognostic factors include stage, capillary lymphatic space invasion, and older age [31].

In most Western countries, the prognosis of patients with vulvar cancer has remained unchanged over the past two to four decades or has increased to a clinically insignificant extent [9]. Although infrequent, data showing a decrease in survival over time have also been published [9]. This disappointing situation is the result of multiple factors common to “orphan” diseases, including the difficulty of recruiting patients for treatment trials, the industry’s lack of interest in developing new effective therapies for small markets, the unavailability of specific screening techniques, the inability of health systems to promote the clinical detection of vulavirus cancers at an earlier stage and the absence of effective networks between primary/secondary health establishments and specialized tertiary centers [9] .

### Limitations

Our study shares common limitations with other retrospective studies. Our study considered only biopsies that were received and processed at the pathological anatomy and cytology laboratory of the CHU Sylvanus Olympio, a functioning healthcare facility in the country. However, it’s important to acknowledge that this laboratory occasionally faces challenges in managing and processing all samples, particularly those originating from more remote regions within the country.

## Conclusion

Vulvar cancer primarily affects postmenopausal women and is considered a rare condition. However, there has been a recent resurgence in cases, likely due to the involvement of the Human Papillomavirus (HPV). (HPV). Squamous cell carcinoma is the most frequently observed histological type, with the ulcero-budding form being predominant. HPV is a significant risk factor, showing a statistically significant association with the histological type of vulvar cancer. Additionally, a familial predisposition to vulvar cancer has been identified as a noteworthy element, potentially facilitating early detection early detection. To reduce the incidence of this cancer, it is recommended to conduct a study on the awareness of vulvar cancer risk factors among the population, thus better equipping them to combat this disease. The implementation of a national HPV vaccination strategy is crucial for improving the management of vulvar cancer cases.

## Data Availability

Extracted data are with the corresponding author and available under reasonable request.

## Abbreviations

CHU: University Hospital Center
EGFR: Human Epidermal Growth Factor receptor
HPV: Human Papilloma Virus
Gy: Gray
HIV: Human Immunodeficiency Virus
VIN: Vulvar Intraepithelial Neoplasia
UICC: Union for International Cancer Control
5-FU: 5-fluorouracil
VEGF: Vascular Endothelial Growth Factor
